# Knock-down the expression of *TaH2B*-*7D* using virus-induced gene silencing reduces wheat drought tolerance

**DOI:** 10.1186/s40659-019-0222-y

**Published:** 2019-03-21

**Authors:** Xinbo Wang, Yongzhe Ren, Jingjing Li, Zhiqiang Wang, Zeyu Xin, Tongbao Lin

**Affiliations:** 1 College of Agronomy, Henan Agricultural University, Zhengzhou, 450002 China; 2 State Key Laboratory of Wheat and Maize Crop Science, Henan Agricultural University, Zhengzhou, 450002 China; 3 Collaborative Innovation Center of Henan Grain Crops, Henan Agricultural University, Zhengzhou, 450002 China

**Keywords:** Drought, *Triticum aestivum* L., *TaH2B*-*7D*, Knock-down

## Abstract

**Background:**

Drought is a major abiotic stress affecting global wheat (*Triticum aestivum* L.) production. Exploration of drought-tolerant genes is essential for the genetic improvement of drought tolerance in wheat. Previous studies have shown that some histone encoding genes are involved in plant drought tolerance. However, whether the *H2B* family genes are involved in drought stress response remains unclear.

**Methods:**

Here, we identified a wheat histone H2B family gene, *TaH2B*-*7D*, which was significantly up-regulated under drought stress conditions. Virus-induced gene silencing (VIGS) technology was used to further verify the function of *TaH2B*-*7D* in wheat drought tolerance. The phenotypic and physiological changes were examined in the *TaH2B*-*7D* knock-down plants.

**Results:**

In the *TaH2B*-*7D* knock-down plants, relative electrolyte leakage rate and malonaldehyde (MDA) content significantly increased, while relative water content (RWC) and proline content significantly decreased compared with those in the non-knocked-down plants under drought stress conditions. *TaH2B*-*7D* knock-down plants exhibited severe sagging, wilting and dwarf phenotypes under drought stress conditions, but not in the non-knocked-down plants, suggesting that the former were more sensitive to drought stress.

**Conclusion:**

These results indicate that *TaH2B*-*7D* potentially plays a vital role in conferring drought tolerance in wheat.

## Background

Drought stress is the principal abiotic factor limiting wheat (*Triticum aestivum* L.) productivity in arid and semi-arid areas [1]. More than 50% of the wheat growing areas in the world are impacted by drought stress [2]. A large number of studies have been carried out on the physiological changes of wheat plants under drought stress and their molecular mechanisms in response to drought stress [3–10]. However, although significant progress has been made [11, 12], the mechanisms of drought tolerance in hexaploid wheat have not been fully explored. Further exploration of drought-tolerant genes is of vital importance for the genetic improvement of wheat drought tolerance.

Studies have shown that the histones are involved in multiple stress responses in plants. Histone proteins contain large amounts of basic amino acids such as arginine and lysine, which are up to about 1/4 of all amino acid residues. The histones proteins bind to the negatively charged double helix DNA to form a chromatin complex [13, 14]. According to the composition of amino acid and molecular weight, histones can be divided into five major families: H_1_, H_2_A, H_2_B, H_3_, H_4_ [15, 16]. Altering the activity or level of histone variants has been demonstrated to be associated with abiotic stress responses [17]. Epigenetic modifications of histone proteins such as deacetylation [18], methylation [19, 20] and ubiquitination [21] are involved in plant drought response. Moreover, knock-down the drought-inducible *H1*-*S* variant of tomato by antisense technology promotes stomatal closure and enhances drought tolerance [17]. The *H2A.Z* variant of *Arabidopsis* is involved in the response to phosphate deficiency [22] as well as in the perception of ambient temperature [23]. Overexpression one of the *TaH2A* variant *TaH2A.7* in *Arabidopsis* significantly lowered water loss rate, and promoted ABA-induced stomatal closure and enhanced drought tolerance in *Arabidopsis* [24]. Histone H2B is one of the four main histone proteins involved in the structure formation of nucleosomes of chromatin in eukaryotic cells [25]. However, whether H2B proteins are involve in the drought stress response is unclear.

Virus-induced gene silencing (VIGS) is an efficient post-transcriptional gene silencing (PTGS)-based technique for gene functional study [26]. It employs the natural defense mechanisms used by plants to protect against invading viruses [27]. Viruses that do not have or have only weak gene silencing suppressors are modified to VIGS systems to induce PTGS-mediated degradation of target plant mRNAs [28–30]. So far, several VIGS systems have been established for monocots [30, 31]. *Barley stripe mosaic virus* (BSMV) is a tripartite RNA virus that can infect many agronomical important crops like barley, wheat, rice, maize and oat, and the BSMV-derived VIGS system has been widely used among monocots [32]. Similarly, *Brome mosaic virus* (BMV) is another RNA virus that has been adopted for VIGS in barley, rice, and maize [33]. The VIGS system developed from the *Rice tungro bacilliform virus* (RTBV) is a convenient and efficient method using agroinoculation, which can reduce the expression levels of target genes by more than 90%. In important horticultural specie orchids, a VIGS vector system has also been successful established employing the symptom free *Cymbidium mosaic virus* (CymMV) [30, 33]. In this study, we identified a drought-responsive histone *H2B* family gene on chromosome 7D, *TaH2B*-*7D*, which was significantly up-regulated under drought stress conditions. As the BSMV-derived VIGS system has been widely used for identification of stress responsive genes in hexaploid wheat [34–38], it was used here to further investigate the function of the drought responsive gene *TaH2B*-*7D*. The phenotypic and physiological changes were examined in the VIGS-based *TaH2B*-*7D* gene knock-down plants. Our results demonstrate that relative electrolyte leakage rate and malonaldehyde (MDA) content significantly increased, while the relative water content (RWC) and proline content significantly decreased in the *TaH2B*-*7D* knock-down plants under drought stress conditions. Moreover, the *TaH2B*-*7D* knock-down plants were more sensitive to drought stress. This work shows that *TaH2B*-*7D* potentially plays a vital role in conferring drought tolerance in common wheat.

## Methods

### Plant material and growth conditions

An elite drought-tolerant wheat variety in China, XN979, was used for in vitro transcribed RNA inoculation in the VIGS trial [39]. Pot culture was employed in the trial. Firstly, seeds were germinated for 16 h at 22 °C; then, twelve germinated seeds were sown in each pot with a soil water content of 90% field capacity (FC). The incubator temperature was set at 21 ± 1 °C in the daytime and 19 ± 1 °C at night (15 h light/9 h dark). Wheat plants were thinned to nine plants per pot after emergence. Sixteen days after sowing, wheat seedling plants (Zadoks growth scale 12) were used for in vitro transcribed RNA inoculation in the VIGS trial. The procedure for vector construction and in vitro transcribed RNA inoculation will be described in detail later. After the inoculation, the pots were divided into two groups and the following two treatments were performed separately: (1) non-stress conditions (NS, maintained the soil water content at 80–90% FC), and (2) drought stress conditions (DS, no watering after sowing). Sixteen days after the inoculation (about 44% FC under DS conditions), the leaves of each pot were collected for measurement of proline and MDA content, RWC and rate of relative electrolyte leakage. In the meanwhile, another trial comprising low nitrogen treatment (LN), salt stress treatment (SS) and non-stressed control were carried out according to previous literatures [40, 41].

### Vector construction and in vitro transcribed RNA inoculation

Vectors for VIGS trial were constructed as previously described [42]. Firstly, a 135 bp-fragment of *TaH2B*-*7D* cDNA coding region was cloned and then inserted into the γ vector (forward primer containing the Pac I restriction site and two protective bases (CC) at 5 prime end: 5′CCTTAATTAAGACAAGAAGAAGAAGAAGGC3′; reverse primer containing the Not I restriction site and three protective bases (TAT) at 5 prime end: 5′TATGCGGCCGCGTCGTTGATGAAGGAGTTC3′). The BSMV_0_ derived from the original empty pSL038-1 vector and acted as a negative control. BSMV_*PDS*_ was used as a positive control to monitor the time course of VIGS [35, 39]. Then, the constructs were linearized and used to synthesize α, β, γ RNAs of the BSMV genome using Ribo MAX TM Large Scale RNA Production System-T7 (Promega, Madison) [43]. The α, β, γ RNAs were mixed in equal amounts and diluted with an equal volume of RNAase-free water and added to FES buffer [34]. Each of the constructs consisted of BSMV α, β, and γ with the *TaH2B*-*7D* gene fragment (BSMV_*TaH2B*-*7D*_) or phytoene desaturase (GenBank: FJ517553.1, BSMV_*PDS*_) or null insertion (BSMV_0_). The inoculation of each viral construct was performed according to previously described procedures [35]. The incubator temperature was set at 23 ± 1 °C, with darkness for 24 h, followed by a 15 h light/9 h dark photoperiod [39].

### Real-time PCR analysis

Leaf total RNA was extracted using Trizol reagent according to the product instructions (Trizol; Invitrogen). Two-Step Prime-Script™ RT reagent Kit with gDNA Eraser (Perfect Real Time; TaKaRa) was used for the cDNA synthesis. The temperature procedure was set as follows: 2 min at 42 °C, 15 min at 37 °C, 5 s at 85 °C, and then 4 °C. The primers used for real-time PCR were designed using Primer 5.0 software (forward primer: 5′GACAAGAAGAAGAAGAAGGC3′; reverse primer: 5′GTCGTTGATGAAGGAGTTC3′). Real-time PCR was performed on a Bio-Rad IQ5 Real-Time PCR Detection System. Each reaction contained 0.4 μmol of forward and reverse primers respectively, 12.5 μl of SYBR Premix Ex Taq (Tli RNaseH Plus), 4 μl diluted cDNA templates. The reaction volume was added to 25 μl with nuclease-free water. The temperature procedure was set as follows: 95 °C for 5 min followed by 40 cycles of 95 °C for 15 s, 60 °C for 15 s, and 72 °C for 15 s. The internal reference gene *TaActin* (forward primer: 5′ ACCTTCAGTTGCCCAGCAAT 3′; reverse primer: 5′ CAGAGTCGAGCACAATACCAGTTG 3′) and *TaGAPDH* (forward primer: 5′ TGTCTGTGGTGTCAATGAGAAGGA 3′; reverse primer: 5′ GCAAGAGGAGCAAGGCAGTTAGT 3′) were used to normalize the expression level of *TaH2B*-*7D*. Three biological replicates were performed. Relative gene expression levels of *TaH2B*-*7D* were calculated using 2^−∆∆CT^ method.

### Measurement of physiological indices

The RWC was measured according to Flexas et al with minor modifications [44]. In brief, fresh leaves were sampled and weighted for fresh weight (FW). Then, the leaves were floated on deionized water for several hours until constant weight to determine their turgid weight (TW). Dry weight (DW) was determined by drying the fully turgid leaves in an oven at 80 °C for several hours until constant weight. The RWC was calculated by using the following formula: RWC (%) = [(FW − DW)/(TW − DW)] × 100. Three independent biological replicates were performed for each measurement.

Electrolyte leakage was measured as described by Yan et al. [45]. Fresh leaves were cut into 10 cm segments and washed three times with ultrapure water. The segments were incubated in a tube containing 10 ml of ultrapure water at room temperature for 24 h. Then, conductivity (C 1) was recorded using a conductivity meter (DDS-307A, China). Subsequently, the tubes were incubated at 100 °C for 20 min. After the solution was cooled to room temperature, conductivity (C 2) was recorded again. Electrolyte leakage was calculated by using the following formula: Electrolyte leakage (%) = C 1/C 2 × 100.

Proline was extracted and determined according to the method of Bates et al. with minor modifications [46]. Firstly, 0.5 g fresh leaves were homogenized in 5 ml 3% (w/v) aqueous sulfosalicylic acid. After centrifuged at 3000×*g* for 15 min at 4 °C, the supernatant was treated with equal volume of acid ninhydrin and glacial acetic acid, and boiled at 100 °C for 20 min, then placed on ice for 10 min. The absorbance of reaction mixture was recorded at 520 nm. Proline content was determined by a standard curve and calculated based on fresh weight (μg g FW^−1^).

MDA content was measured according to the method of Hodges et al. with minor modifications [47]. In brief, 0.5 g fresh leaves were sampled and fast-frozen in liquid nitrogen. Then the samples were fully grinded using a tissue grinder. 5 ml of 5% (w/v) trichloroacetic acid (TCA) was added to each sample and mixed thoroughly. The mixture was centrifuged at 4 °C, 4000×*g* for 20 min, and 1 ml of supernatant was transfer to equal volume of 0.5% (v/v) TBA in 20% TCA. The mixture was boiled at 100 °C for 30 min, and then placed on ice for 30 min. After centrifuged at 4000×*g* for 10 min at 4 °C, the absorbance of 2 ml supernatant was recorded at 450 nm, 532 nm and 600 nm, respectively. MDA content was calculated by using the following formula: MDA content (μmol g FW^−1^) = (6.45 (OD_532 _− OD_600_) − 0.56 OD_450_) × V/W. In the formula, V represents the volume of extracts (5 ml) and W represents the fresh weight of sample (0.5 g).

## Results

### The expression of *TaH2B*-*7D* under NS and DS conditions

**Fig. 1:**
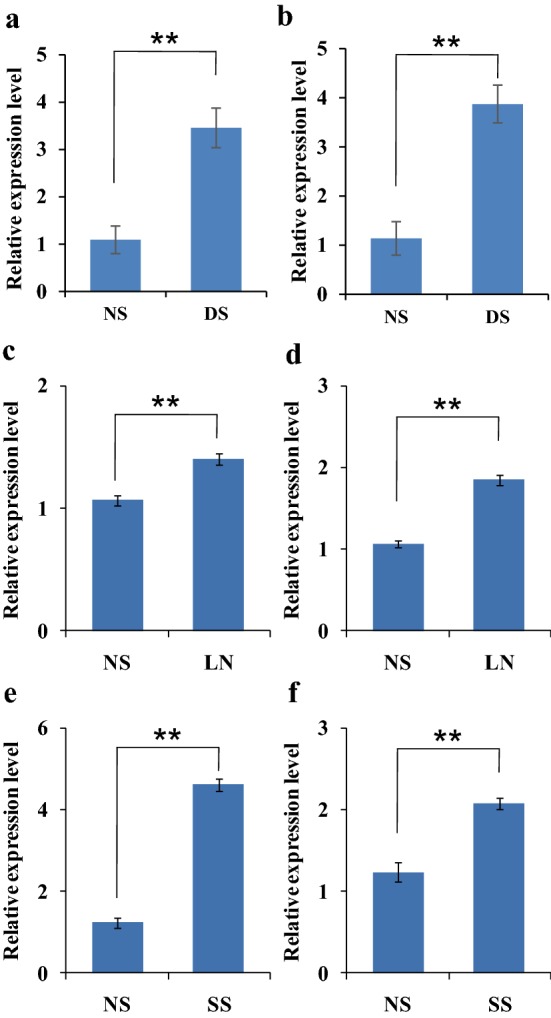
Relative expression level of *TaH2B*-*7D* under drought stress, low nitrogen and salt stress conditions. **a**, **b** drought stress; **c**, **d** low nitrogen stress; **e**, **f** salt stress. **a**, **c**, **e** Real-time PCR analysis of *TaH2B*-*7D* using *TaActin* as reference gene. **b**, **d**, **f** Real-time PCR analysis of *TaH2B*-*7D* using *TaActin* as reference gene. *NS* non-stress; *DS* drought stress; *LN* low nitrogen; *SS* salt stress. Each bar shows the mean ± standard errors (SE). Double asterisk indicate significant differences at *P* ≤ 0.01 levels

**Fig. 2:**
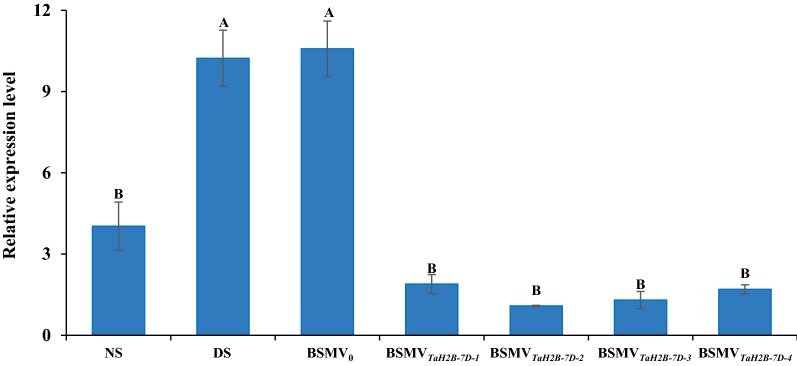
Detection of the expression levels of *TaH2B*-*7D* in the gene knock-down and control plants. NS, non-stress; DS, drought stress. BSMV_0_, negative control of the VIGS system; BSMV_*TaH2B*-*7D*_, *TaH2B*-*7D*-knock down plants; BSMV_*TaH2B*-*7D*-*1*_, BSMV_*TaH2B*-*7D*-*2*_, BSMV_*TaH2B*-*7D*-*3*_ and BSMV_*TaH2B*-*7D*-*4*_ are four independent *TaH2B*-*7D*-knock down plants. The expression level of *TaH2B*-*7D* in the *TaH2B*-*7D*-knock down plants was detected under DS conditions. Different letters above the columns indicate significant differences at *P* ≤ 0.01 levels

Firstly, we examined the expression of *TaH2B*-*7D* under non-stress (NS) and drought stress (DS) conditions. Result shows that the expression of *TaH2B*-*7D* in XN979 was significantly up-regulated by drought stress (Fig. 1a, b). Since previous studies have shown that histones are involved in multiple stress responses, we examined the expression of *TaH2B*-*7D* under low nitrogen and salt stress conditions. Results show that *TaH2B*-*7D* was also significantly up-regulated by low nitrogen stress and salt stress (Fig. 1c–f). To check the effect of VIGS in our study, the expression level of *TaH2B*-*7D* was investigated in four independent BSMV_*TaH2B*-*7D*_-infected plants (BSMV_*TaH2B*-*7D*-*1*_, BSMV_*TaH2B*-*7D*-*2*_, BSMV_*TaH2B*-*7D*-*3*_ and BSMV_*TaH2B*-*7D*-*4*_) and controls. Results show that the expression of *TaH2B*-*7D* were significantly down-regulated in the BSMV_*TaH2B*-*7D*_-infected plants compared with that in the non-infected and BSMV_0_-infected plants (negative control) under DS conditions (Fig. 2), indicating that the expression levels of *TaH2B*-*7D* have been successfully knocked-down in the BSMV_*TaH2B*-*7D*_-infected individuals.

### Phenotypic changes in the *TaH2B*-*7D* knock-down plants

**Fig. 3:**
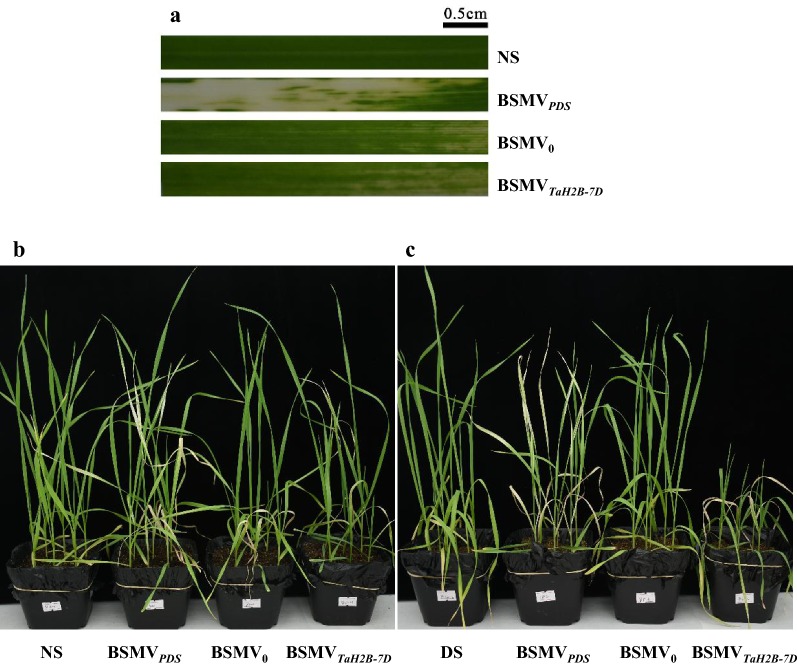
The phenotypes of *TaH2B*-*7D*-knock down plants. **a** Leaf; **b**, **c** whole plants; **b** non-stress treatment (NS); **c** drought stress treatment (DS). BSMV_0_ represents the negative control of VIGS system; BSMV_*PDS*_ represents the positive control monitoring time course of VIGS; BSMV_*TaH2B*-*7D*_ represents *TaH2B*-*7D*-knock down plants

In the VIGS trial, 10 days after inoculation, all the BSMV constructs-infected plants exhibited slight chlorosis owing to the plant immunity to virus. The BSMV_*PDS*_-infected plants emerged visible bleached leaves (Fig. 3a), indicating the success of the viral inoculation [39]. Twenty days after inoculation, there were no obvious phenotypic changes of BSMV_*TaH2B*-*7D*_-infected plants under NS conditions compared with the non-infected and BSMV_0_-infected plants (Fig. 3b). However, severe leave sagging, wilting and slow growth (dwarf) were presented in the BSMV_*TaH2B*-*7D*_-infected plants under DS conditions (Fig. 3c).

### Physiological changes of the *TaH2B*-*7D* knock-down plants

**Fig. 4:**
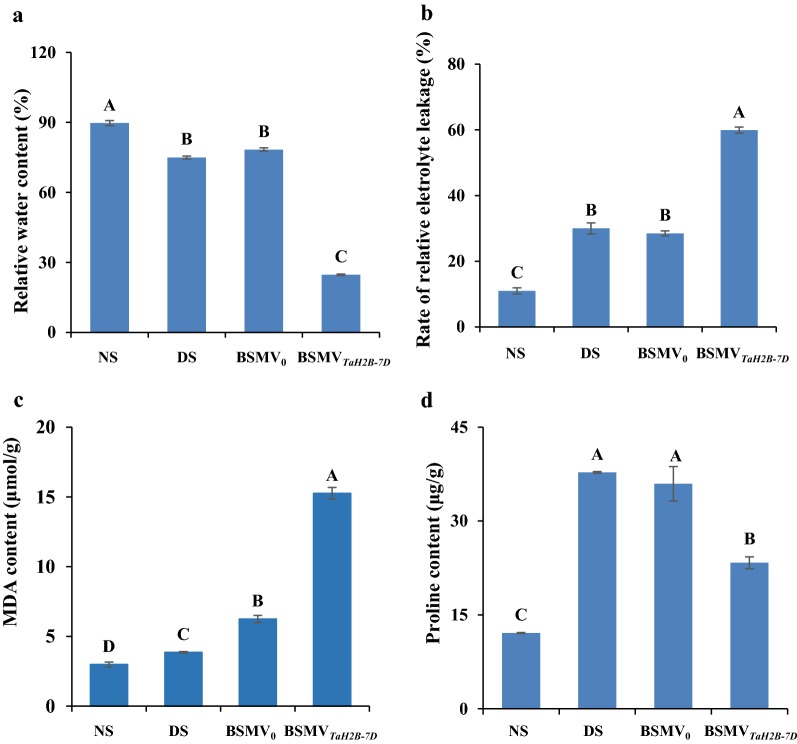
The changes in the physiological indices of the *TaH2B*-*7D*-knock-down plants. **a** Relative water content; **b** rate of relative electrolyte leakage; **c** MDA content; **d** proline content. NS, non-stressed plants; DS, drought-stressed plants; BSMV_0_, negative control of the VIGS system; BSMV_*TaH2B*-*7D*_*, TaH2B*-*7D*-knock-down plants. Each bar shows the mean ± standard errors (SE) for three biological replicates

We also checked physiological changes in the *TaH2B*-*7D* knock-down plants. Under DS conditions, leaf RWC in the non-infected plants and BSMV_0_-infected plants only decreased by 16.4% and 14.5%, respectively, compared with that in the NS non-infected plants. However, leaf RWC in the BSMV_*TaH2B*-*7D*_-infected plants under DS conditions reduced by 67.0% compared with that in the NS non-infected plants (Fig. 4a). At the same time, relative electrolyte leakage rate in the BSMV_*TaH2B*-*7D*_-infected plants increased by 446.2% under DS conditions compared with that in the NS non-infected plants, which was significantly higher than that in the non-infected (173.5%) and BSMV_0_-infected (159.6%) plants under DS conditions (Fig. 4b). Moreover, MDA content in the BSMV_*TaH2B*-*7D*_-infected plants increased by 410.4% under DS conditions compared with that in the NS non-infected plants, which was also significantly higher than that in the non-infected and BSMV_0_-infected individuals (negative controls) under DS conditions (Fig. 4c). In addition, proline content of the BSMV_*TaH2B*-*7D*_-infected plants under DS conditions increased by 93% compared with that in the NS non-infected plants, which is obvious lower than was the case in both non-infected (211.8%) and BSMV_0_-infected (196.9%) individuals (Fig. 4d).

## Discussion

Plants inevitably come across complicated environmental changes during their life cycle. Drought is one of the major limiting factors for plant growth and productivity [48, 49]. Identification of drought tolerance-related genes is very important for the genetic improvement of plant drought tolerance. Currently, many drought-responsive genes/proteins have been identified in different species such as wheat, maize, rice, peanut and soybean in previous studies [50–55]. These results are of great significance for exploring the molecular mechanisms and genetic improvement of wheat drought tolerance [56, 57]. However, most of these genes/proteins have not been functionally verified, especially in hexaploid wheat. Gene functional verification by genetic transformation in wheat is time-consuming and high-cost. Verification the functions of the large number of drought responsive genes/proteins is a big challenge in hexaploid wheat. VIGS technology is an alternative approach for preliminary functional analysis of these genes/proteins because of its rapidity and high efficiency [34–38]. In this study, VIGS was used to further analysis the function of a drought stress up-regulated histone H2B family gene, *TaH2B*-*7D*.

Histone proteins have been proved to be involved in multiple stress response [17]. For example, some histone protein variants are involved in the response to low phosphate and drought stress response, and temperature perception [17, 22, 23]. Moreover, deacetylation, methylation and ubiquitination of histone proteins are also involved in plant drought response [18–21]. A recent study showed that overexpression *Arabidopsis AtHUB2* gene in cotton increases the global H2B monoubiquitination (H2Bub1) level through a direct interaction with GhH2B1 and up-regulates the expression of drought-related genes in transgenic cotton plants [21]. Coincidentally, the expression level of *TaH2B*-*7D* was also up-regulated by drought stress in wheat (Fig. 1a, b). The evidences indicate that H2B proteins may play a role in plant drought stress response. Since the expression of *TaH2B*-*7D* was significantly up-regulated by DS (Figs. 1, 2), VIGS as a post-transcriptional gene silencing technology, is suitable for the functional study of this gene In the VIGS trial, we observed a significant decrease of *TaH2B*-*7D* expression level in all the four independent BSMV_*TaH2B*-*7D*_-infected lines, indicating that the expression level of *TaH2B*-*7D* was efficiently knocked-down (Fig. 2). Conventionally, the degree of leaf drooping is less, and leaves can maintain a relatively higher RWC in drought-tolerance plants than drought-sensitive individuals under DS conditions [58, 59]. Thus, the RWC of plant leaves can be used to at least partially assess the drought tolerance of a plant. In the *TaH2B*-*7D* knocked-down plants, leaf RWC significantly decreased compared with that in the negative control (BSMV_0_-infected plants) under DS conditions (Fig. 4a). This result indicates that *TaH2B*-*7D* knock-down plants are more sensitive to drought stress. MDA content is an important marker of structural damage of the membrane [60]. Previous studies have shown that when plants are subjected to severe drought stress, the membrane is easily broken, membrane proteins are damaged, and the relative conductivity and MDA are significantly increased [61, 62]. In this study, the relative electrolyte leakage rate and MDA content in the *TaH2B*-*7D* knocked-down plants were both significantly higher than those in the non-infected and BSMV_0_-infected plants under DS conditions (Fig. 4b, c). Moreover, compared with the NS plants, the increased ratio of proline content in the BSMV_*TaH2B*-*7D*_-infected plants was significantly lower than that in the non-infected control and BSMV_0_-infected plants under DS conditions. Proline is an important osmoregulatory substance that exists in plant cells. Previous studies have shown that the accumulation of proline favors osmotic adjustment and cell membrane stabilization under DS conditions [63, 64]. Taken together, these physiological results further confirm that *TaH2B*-*7D* knock-down plants are more sensitive to drought stress, indicating that *TaH2B*-*7D* potentially plays a vital role in conferring drought tolerance in hexaploid wheat.

In this study, we did not find any significant phenotypic change of *TaH2B*-*7D* knock-down plants under NS conditions. This result is beyond our previous expectation. After all, histones are essential components of chromatin. However, we had repeated this experiment two more times and got similar results. One possible reason to explain this result is that different members of H2B family genes may have a more meticulous functional differentiation. Some of them are essential components of chromatin under normal growth conditions, while others may play roles when plants encounter various environmental stresses. Interestingly, the expression level of *TaH2B*-*7D* was up-regulated not only by drought stress, but also by low nitrogen and salt stresses (Fig. 1). These results indicate that *TaH2B*-*7D* may play important roles in responding to multiple abiotic stresses. In this study, although the gene-specific primers of *TaH2B*-*7D* were designed and the sequence of the inserted cDNA fragment was confirmed by Sanger sequence when constructing the VIGS vectors, the possibility of knocking down some homologous genes of *TaH2B*-*7D* could not be completely ruled out. Therefore, further studies are needed to generate transgenic lines that overexpress and/or underexpress *TaH2B*-*7D* to better understand the function of this gene.

## Conclusion

Knock-down the expression level of *TaH2B*-*7D* in wheat plants significantly increased leaf relative electrolyte leakage rate and MDA content, decreased leaf RWC and proline accumulation, and reduced wheat drought tolerance. Therefore, *TaH2B*-*7D* potentially plays a vital role in conferring drought tolerance in hexaploid wheat.

## Abbreviations

VIGS: virus-induced gene silencing
MDA: malonaldehyde
RWC: relative water content
DS: drought stress
NS: no stress
FC: field capacity
PTGS: post-transcriptional gene silencing
BSMV: barley stripe mosaic virus
BMV: brome mosaic virus
RTBV: rice tungro bacilliform virus
CymMV: cymbidium mosaic virus
LN: low nitrogen
SS: salt stress

## References

[1] Zadražnik T, Hollung K, Egge-jacobsen W, Meglič V, Šuštar-Vozlič J (2013). Differential proteomic analysis of drought stress response in leaves of common bean (*Phaseolus vulgaris* L.). J Proteome..

[2] Rajaram S (2001). Prospects and promise of wheat breeding in the 21st century. Euphytica.

[3] Lopez CG, Banowetz GM, Peterson CJ, Kronstad WE (2003). Dehydrin expression and drought tolerance in seven wheat cultivars. Crop Sci.

[4] Fleury D, Jefferies H, Kuchel H, Langridge P (2010). Genetic and genomic tools to improve drought tolerance in wheat. J Exp Bot.

[5] Miller GAD, Suzuki N, Ciftci-Yilmaz S, Mittler RON (2010). Reactive oxygen species homeostasis and signalling during drought and salinity stresses. Plant Cell Environ.

[6] Kosová K, Vítámvás P, Prášil IT (2014). Proteomics of stress responses in wheat and barley-search for potential protein markers of stress tolerance. Front Plant Sci..

[7] Ferdous J, Hussain SS, Shi BJ (2015). Role of microRNAs in plant drought tolerance. Plant Biotechnol J.

[8] Gahlaut V, Jaiswal V, Kumar A, Gupta PK (2016). Transcription factors involved in drought tolerance and their possible role in developing drought tolerant cultivars with emphasis on wheat (*Triticum aestivum* L.). Theor Appl Genet.

[9] Botha AM, Kunert KJ, Cullis CA (2017). Cysteine proteases and wheat (*Triticum aestivum* L) under drought: a still greatly unexplored association. Plant, Cell Environ.

[10] Saradadevi R, Palta JA, Siddique KHM (2017). ABA-mediated stomatal response in regulating water use during the development of terminal drought in wheat. Front Plant Sci..

[11] Singh S, Gupta AK, Kaur N (2012). Differential responses of antioxidation defence system to long-term filed drought in wheat (*Triticum aestivum* L.) genotypes differing in drought tolerance. J Agron Crop Sci.

[12] Ouyang WJ, Struik PC, Yin XY, Yang JC (2017). Stomatal conductance, mesophyll conductance, and transpiration efficiency in relation to leaf anatomy in rice and wheat genotypes under drought. J Exp Bot.

[13] Annunziato A (2008). DNA packaging: nucleosomes and chromatin. Nature Education..

[14] Kornberg RD (1974). Chromatin structure: a repeating unit of histones and DNA. Science.

[15] Luger K, Mader AW, Richmond RK, Sargent DF, Richmond TJ (1997). Crystal structure of the nucleosome core particle at 28 Å resolution. Nature.

[16] Mariño-Ramírez L, Kann MG, Shoemaker BA, Landsman D (2005). Histone structure and nucleosome stability. Expert Rev Proteomics..

[17] Scippa GS, Di Michele M, Onelli E, Patrignani G, Chiatante D, Bray EA (2004). The histone-like protein H1-S and the response of tomato leaves to water deficit. J Exp Bot.

[18] Kim JM, To TK, Ishida J, Matsui A, Kimura H, Seki M (2012). Transition of chromatin status during the process of recovery from drought stress in *Arabidopsis thaliana*. Plant Cell Physiol.

[19] Fu YL, Zhang GB, Lv XF, Guan Y, Yi HY, Gong JM (2013). *Arabidopsis* histone methylase CAU1/PRMT5/SKB1 acts as an epigenetic suppressor of the calcium signaling gene CAS to mediate stomatal closure in response to extracellular calcium. Plant Cell..

[20] Fu Y, Ma H, Chen S, Gu T, Gong J (2017). Control of proline accumulation under drought via a novel pathway comprising the histone methylase CAU1 and the transcription factor ANAC055. J Exp Bot.

[21] Chen H, Feng H, Zhang XY, Zhang CJ, Wang T, Dong JL (2018). An *Arabidopsis* E3 ligase HUB2 increases histone H2B monoubiquitination and enhances drought tolerance in transgenic cotton. Plant Biotechnol J.

[22] Smith AP, Jain A, Deal RB, Nagarajan VK, Poling MD, Raghothama KG, Meagher RB (2010). Histone H2A.Z regulates the expression of several classes of phosphate starvation response genes but not as a transcriptional activator. Plant Physiol.

[23] Kumar SV, Wigge PA (2010). H2A.Z-containing nucleosomes mediate the thermosensory response in *Arabidopsis*. Cell.

[24] Xu W, Li Y, Cheng Z, Xia G, Wang M (2016). A wheat histone variant gene TaH2A.7 enhances drought tolerance and promotes stomatal closure in *Arabidopsis*. Plant Cell Rep.

[25] Bhasin M, Reinherz EL, Reche PA (2006). Recognition and classification of histones using support vector machine. J Comput Biol.

[26] Baulcombe DC (1999). Fast forward genetics based on virus-induced gene silencing. Curr Opin Plant Biol.

[27] Voinnet O (2001). RNA silencing as a plant immune system against viruses. Trends Genet.

[28] Li WX, Ding SW (2001). Viral suppressors of RNA silencing. Curr Opin Biotechnol.

[29] Cao X, Zhou P, Zhang X, Zhu S, Zhong X, Xiao Q, Ding B, Li Y (2005). Identification of an RNA silencing suppressor from a plant double-stranded RNA virus. J Virol.

[30] Ramegowda V, Mysore KS, Senthil-Kumar M (2014). Virus-induced gene silencing is a versatile tool for unraveling the functional relevance of multiple abiotic-stress-responsive genes in crop plants. Front Plant Sci..

[31] Gutierrez C (2002). Strategies for geminivirus DNA replication and cell cycle interference. Physiol Mol Plant Pathol.

[32] Pacak A, Geisler K, Jørgensen B, Barciszewska-Pacak M, Nilsson L, Nielsen TH, Johansen E, Grønlund M, Jakobsen I, Albrechtsen M (2010). Investigations of barley stripe mosaic virus as a gene silencing vector in barley roots and in *Brachypodium distachyon* and oat. Plant Methods..

[33] Lange M, Yellina AL, Orashakova S, Becker A (2013). Virus-induced gene silencing (VIGS) in plants: an overview of target species and the virus-derived vector systems. Methods Mol Biol.

[34] Scofield SR, Huang L, Brandt AS, Gill BS (2005). Development of a virus-induced gene-silencing system for hexaploid wheat and its use in functional analysis of the Lr21-mediated leaf rust resistance pathway. Plant Physiol.

[35] Zhou H, Li S, Deng Z, Wang X, Chen T, Zhang J, Chen S, Ling H, Zhang A, Wang D (2007). Molecular analysis of three new receptor-like kinase genes from hexaploid wheat and evidence for their participation in the wheat hypersensitive response to stripe rust fungus infection. Plant J..

[36] Van Eck L, Schultz T, Leach JE, Scofield SR, Peairs FB, Botha AM, Lapitan NL (2010). Virus-induced gene silencing of WRKY53 and an inducible phenylalanine ammonia-lyase in wheat reduces aphid resistance. Plant Biotechnol J.

[37] Scofield SR, Brandt AS (2012). Virus-induced gene silencing in hexaploid wheat using barley stripe mosaic virus vectors. Methods Mol Biol.

[38] Zhang N, Huo W, Zhang L, Chen F, Cui D (2016). Identification of winter-responsive proteins in bread wheat using proteomics analysis and virus-induced gene silencing (VIGS). Mol Cell Proteom.

[39] Wang X, Xu Y, Li J, Ren Y, Wang Z, Xin Z, Lin T (2018). Identification of two novel wheat drought tolerance-related proteins by comparative proteomic analysis combined with virus-induced gene silencing. Int J Mol Sci.

[40] Ren YZ, Xu YH, Teng W, Li B, Lin TB (2018). QTLs for seedling traits under salinity stress in hexaploid wheat. Ciencia Rural.

[41] Ren YZ, Yue HF, Li L, Xu YH, Wang ZQ, Xin ZY, Lin TB (2018). Identification and characterization of circRNAs involved in the regulation of low nitrogen-promoted root growth in hexaploid wheat. Biol Res.

[42] Cakir C, Scofield S (2008). Evaluating the ability of the barley stripemosaic virus-induced gene silencing system to simultaneously silence two wheat genes. Cereal Res Commun..

[43] Petty IT, Hunter BG, Wei N, Jackson AO (1989). Infectious barley stripe mosaic virus RNA transcribed in vitro from full-length genomic cDNA clones. Virology.

[44] Flexas J, Ribas-Carbó M, Bota J, Galmés J, Henkle M, Martínez-Cañellas S, Medrano H (2006). Decreased Rubisco activity during water stress is not induced by decreased relative water content but related to conditions of low stomatal conductance and chloroplast CO_2_ concentration. New Phytol.

[45] Yan SP, Zhang QY, Tang ZC, Sun WN (2006). Comparative proteomic analysis provides new insights into chilling stress responses in rice. Mol Cell Proteom.

[46] Bates LS, Waldren RP, Teare ID (1973). Rapid determination of free proline for water stress studies. Plant Soil.

[47] Hodges DM, DeLong JM, Forney CF, Prange RK (1999). Improving the thiobarbituric acid-reactive-substances assay for estimating lipid peroxidation in plant tissues containing anthocyanin and other interfering compounds. Planta.

[48] Barnabás B, Jäger K, Fehér A (2008). The effect of drought and heat stress on reproductive processes in cereals. Plant Cell Environ.

[49] Baek W, Lim S, Lee SC (2016). Identification and functional characterization of the pepper *CaDRT1* gene involved in the ABA-mediated drought stress response. Plant Mol Biol.

[50] Hajheidari M, Eivazi A, Buchanan BB, Wong JH, Majidi I, Salekdeh GH (2007). Proteomics uncovers a role for redoxin drought tolerance in wheat. J Proteome Res.

[51] Govind G, ThammeGowda HV, Kalaiarasi PJ, Iyer DR, Muthappa SK, Nese S, Makarla UK (2009). Identification and functional validation of a unique set of drought induced genes preferentially expressed in response to gradual water stress in peanut. Mol Genet Genomics.

[52] Krishnan HB, Nelson RL (2011). Proteomic analysis of high protein soybean (*Glycine max*) accessions demonstrates the contribution of novel glycinin subunits. J Agric Food Chem.

[53] Zhao FY, Zhang DY, Zhao YL, Wang W, Yang H, Tai FJ (2016). The difference of physiological and proteomic changes in maize leaves adaptation to drought, heat, and combined both stresses. Front Plant Sci..

[54] Nutwadee C, Maiporn N, Narumon P, Michael VM, Sittiruk R, Supachitra C (2017). Proteomic analysis of drought-responsive proteins in rice reveals photosynthesis-related adaptations to drought stress. Acta Physiol Plant.

[55] Wang X, Komatsu S (2018). Proteomic approaches to uncover the flooding and drought stress response mechanisms in soybean. J Proteom.

[56] Ateş Sönmezoğlu Ö, Terzi B (2018). Characterization of some bread wheat genotypes using molecular markers for drought tolerance. Physiol Mol Biol..

[57] Rampino P, De Pascali M, De Caroli M, Luvisi A, De Bellis L, Piro G, Perrotta C (2017). Td4IN2: a drought-responsive durum wheat (*Triticum durum Desf.*) gene coding for a resistance like protein with serine/threonine protein kinase, nucleotide binding site and leucine rich domains. Plant Physiol Bioch..

[58] Bijanzadeh E, Emam Y (2010). Effect of defoliation and drought stress on yield components and chlorophyll content of wheat. Pak J Biol Sci.

[59] Zegaoui Z, Planchais S, Cabassa C, Djebbar R, Belbachir OA, Carol P (2017). Variation in relative water content, proline accumulation and stress gene expression in two cowpea landraces under drought. J Plant Physiol.

[60] Shao HB, Liang ZS, Shao MA (2005). Changes of anti-oxidative enzymes and MDA content under soil water deficits among 10 wheat (*Triticum aestivum* L.) genotypes at maturation stage. Colloid Surface..

[61] Kocheva KV, Landjeva SP, Georgiev GI (2014). Variation in ion leakage parameters of two wheat genotypes with different Rht-B1 alleles in response to drought. J Biosci.

[62] Demirevska K, Simova-Stoliiva L, Vassileva V, Feller U (2008). Rubisco and some chaperone protein responses to water stress and rewatering at early seedling growth of drought sensitive and tolerant wheat varieties. Plant Growth Regu..

[63] Ashraf M, Foolad M (2007). Roles of glycine betaine and proline in improving plant abiotic stress resistance. Environ Exp Bot.

[64] Yooyongwech S, Samphumphuang T, Tisarum R, Theerawitaya C, Cha-Um S (2017). Water-deficit tolerance in sweet potato [*Ipomoea batatas (L.)* Lam.] by foliar application of paclobutrazol: role of soluble sugar and free proline. Front Plant Sci..

